# Examining the Perspectives and Attitudes Towards Antibiotic Use and Resistance Among the General Public in Muzaffarpur in Bihar, India

**DOI:** 10.7759/cureus.53938

**Published:** 2024-02-09

**Authors:** Soofia Firdaus, FNU Sadaf, Sushant K Sharma, Vishal Prakash, Md Khalid Tanweer, Tauseef Kibria

**Affiliations:** 1 Department of Microbiology, Sheikh Bhikhari Medical College, Hazaribagh, IND; 2 Department of Pathology, Nalanda Medical College, Patna, IND; 3 Department of General Surgery, Sri Krishna Medical College, Muzaffarpur, IND; 4 Department Of Ophthalmology, Nalanda Medical College, Patna, IND

**Keywords:** perception, knowledge, attitude, antibiotic resitance, antibiotic usage

## Abstract

Objective

This study aimed to examine the current understanding and perspectives about the use of antibiotics among the general public living in the city of Muzaffarpur, Bihar, and the surrounding areas.

Methodology

A cross-sectional study was carried out from March 2023 to August 2023 in Muzaffarpur. Data was obtained through interviews based on a standardized questionnaire derived from a prior study. The findings were summarized using descriptive statistics, frequencies, and percentages, and then presented in tabular form.

Results

This study recruited 384 participants in total; the majority of them (n=200, 52.1%) were females, while the remaining 184 (47.9%) participants were males. Our findings revealed that 368 (96%) participants agreed on the necessity of prescribing distinct antibiotics for the treatment of various ailments. Nevertheless, the participants held divergent perspectives regarding the efficacy of antibiotics in treating coughs and colds, as well as their effectiveness against viruses and bacteria. Overall, 354 (92.1%) participants agreed with the importance of finishing the prescribed antibiotic regimen, and 335 (87.2%) agreed that people should refrain from retaining drugs for future use. Of note, 90% of the participants (n=346) stated that they did not think it was advisable to get antibiotics from friends and family without first consulting a doctor.

Conclusion

The current study documented a prevalent utilization of antibiotics among the study participants, with a significant proportion of these medications being obtained without a prescription. The respondents demonstrated a general lack of understanding, particularly concerning the significance of antibiotics in treating mild viral illnesses.

## Introduction

The prevention and treatment of infectious diseases have been significantly impacted by the introduction of antibiotics. Using these therapies in both preventative and curative care has improved overall patient care and enabled the survival of many patients [1]. However, the emergence of bacteria with established resistance seriously jeopardizes the efficiency of therapeutic therapies [2]. Empirical evidence has revealed that infections caused by resistant organisms are linked to prolonged therapy, extended hospital admissions, elevated mortality rates, as well as escalated utilization of supplementary medications, laboratory testing, and other resources, all of which contribute to increased treatment expenditures [3].

A multitude of intricate factors contribute to the emergence of antibiotic resistance. Several complexes are associated with the emergence of antimicrobial resistance. Resistance to bacteria is influenced by the closer integration of the global population and the process of evolution. Yet, it is important to acknowledge the serious consequences of overusing unnecessary antibiotics [4,5]. Antibiotic abuse can linked to a complicated interplay of many causes, including the prescribing practices and knowledge of doctors, diagnostic uncertainty, patient demand, ineffective communication between patients and doctors, and more general factors at the societal, cultural, economic, and policy levels of healthcare [5]. The emergence and spread of antibiotic-resistant bacteria have also been linked to the knowledge, beliefs, attitudes, expectations, and prior experiences of patients [6]. Evidence has shown that patients engage in inappropriate utilization of antibiotics, as exemplified by their lack of compliance with approved treatment plans, failure to adhere to prescribed dosing regimens, repurposing of remaining medications, self-administration of antibiotics, and inappropriate use of antibiotics to treat viral infections [2].

Self-medication, done properly, can benefit patients and the healthcare industry as a whole. According to different studies conducted in various contexts, the proportion of people who self-medicate ranges from 38% to 83%. However, several studies have repeatedly shown that this behavior is quite prevalent. Moreover, the correlation between self-medication and improper drug use has been consistently observed in these studies [7-10]. In the event of an escalation in self-medication utilization, the efficient allocation of resources may be compromised, leading to the emergence of pathogen resistance, the occurrence of adverse reactions, inaccurate self-diagnosis, delayed access to adequate healthcare, and a higher likelihood of dependence and substance abuse [11,12]. One prevalent and significant factor that leads to the development of antibiotic resistance is the use of antibiotics without a doctor's guidance or recommendation [13,14]. For instance, a study carried out among students at a University in Karachi found that about 47.6% of participants admitted to using antibiotics within six months before the study without a legitimate prescription from a doctor [15]. A higher use of antibiotics has also been associated with ignorance and unfavorable attitudes towards the use of antibiotics for self-medication [16,17].

The aforementioned issue could get even worse in countries/regions characterized by inadequate regulatory frameworks, a notable incidence of infectious diseases, and substandard healthcare infrastructure. Furthermore, there is scarce research on community attitudes and awareness regarding antibiotics, and studies done so far have revealed a general lack of information and unfavorable views [17]. To our knowledge, no such research has been conducted among the people who live in Muzaffarpur in Bihar and its surrounding areas. Hence, it is of utmost importance to ascertain the understanding and attitudes of communities about the utilization of antibiotics. This research aims to examine the current understanding and perspectives among the general public living in the city of Muzaffarpur and its adjacent areas about the use of antibiotics.

## Materials and methods

This was a cross-sectional study carried out in the city of Muzaffarpur between March 2023 and August 2023. During the study period, the city had a total of 16 pharmacies and 44 drug retailers. The study included everyone seeking services from certain privately owned retail medical shops. Healthcare practitioners were excluded. The researchers utilized a stratified sampling methodology to ensure the inclusion of a comprehensive and representative sample of drug retail establishments, comprising both pharmacies and drug stores. A non-systematic sampling approach was employed to choose four pharmacies and 10 drug shops. The data on the average influx of customers at each specified pharmaceutical retail shop was used to determine the total sample. Study participants were identified via random sampling. This procedure entailed calculating a precise K-value by dividing the total expected number of consumers over the study period by the required sample size.

The single-population sampling approach was used to determine the sample size for this study. The calculation incorporated a p-value of 50% to represent the prevalence of antibiotic consumption within the past year. Additionally, a 95% confidence interval and a 5% margin of error were taken into account. Using the above criteria, the total sample determined size was 384. The data were obtained through a partial interview process by utilizing a standardized questionnaire derived from a prior study [18], which was modified to align with the specific objectives of our research. To evaluate the reliability and validity of the research instruments, a pilot study was conducted among a subset comprising 5% of the total sample population. Two individuals who collected the data were identified as data collectors and were completing their pharmacy education. Training was provided to data collectors regarding the content of data collection instruments and the interview methodology.

The questionnaires consisted of four components. The primary focus of the first segment was the sociodemographic details, including age, gender, marital status, level of education, occupation, and monthly income. The second portion included nine questions to assess community-wide antibiotic usage patterns. Eight questions were included in the third section to gauge the participants' knowledge of antibiotics. The fourth and last segment contained seven questions to determine how people felt about antibiotics. The survey accepted "yes," "no," and "I don't know" as responses. The response option for attitude questionnaires was created using the 5-point Likert scale, which had the following options: "strongly disagree," "disagree," "neutral," "agree," and "strongly agree." The questionnaires regarding the use of antibiotics had nine questions with several "yes" and "no" response options. These were intended to be closed-ended inquiries with prepared response options. The questionnaires included several questions, such as whether participants had received an antibiotic prescription the year before the research, the number of prescriptions they had, how often they had followed these prescriptions, and, if not, why they had not. We also assessed the sources from where people received their antibiotics and the practice of using antibiotics for self-medication.

Finally, a review of antibiotic-sharing practices was done. SPSS Statistics for Windows version 20 (IBM Corp., Armonk, NY) was employed to evaluate the data after entering it into EpiData 3.1. To summarize the findings, descriptive statistics, frequencies, and percentages were used, which were presented in tabular format.

## Results

This cross-sectional descriptive study recruited a total of 384 participants; the majority of them (n=200, 52.1%) were females, while the remaining 184 (47.9%) were males. A significant proportion of the participants (n=211, 68.49%) fell in the age range of 18-34 years. Conversely, a smaller percentage of the participants (n=129, 33.6%) possessed a high level of education. Of note, 283 (73.7%) participants were living in urban settings at the time of the study. The majority of the participants were merchants (n=92, 24%) while government employment was the source of income for 21.6% of participants (Table 1).

**Table 1:** Demographic characteristics

Variable	N (%)
Age group, years	
≥65	33 (8.6%)
35-64	88 (22.9%)
18-34	263 (68.5%)
Sex	
Female	200 (52.1%)
Male	184 (47.9%)
Marital status	
Single	120 (31.3%)
Married	264 (68.7%)
Residence	
Rural	101 (26.3%)
Urban	283 (73.7%)
Occupation	
Daily laborer	22 (5.7%)
Merchant	92 (24%)
Farmer	57 (14.8%)
Government employee	83 (21.6%)
Housewife	70 (18.3%)
Student	54 (14%)
Others	6 (1.7%)
Education	
Illiterate	51 (13.3%)
Primary education	111 (28.9%)
High school	93 (24.2%)
University or professional degree	129 (33.6%)

As presented in Table 2, the current investigation revealed that 368 (96%) participants agreed on the necessity of prescribing distinct antibiotics for the treatment of various ailments. Nevertheless, the participants held divergent perspectives regarding the efficacy of antibiotics in treating coughs and colds, as well as their effectiveness against viruses and bacteria. Overall, 319 (83%) participants indicated that antibiotics enhanced the recuperation process for the majority of coughs and colds. Meanwhile, 369 (90.1%) participants agreed on the efficacy of antibiotics against bacteria, but slightly fewer than half asserted that antibiotics worked well to combat viruses (n=179, 47%). Promisingly, the study cohort exhibited a higher level of awareness of antibiotic resistance; 301 (78.4%) participants agreed with the notion that the overutilization of antibiotics can contribute to the development of bacterial resistance (Table 2).

**Table 2:** Knowledge of and perceptions toward antibiotic usage

	Yes, n (%)	No, n (%)	I don’t know, n (%)
The issue of antibiotic resistance is a global concern	121 (31.5%)	134 (34.9%)	129 (33.6%)
Various types of antibiotics are required to effectively treat certain diseases	369 (96%)	6 (1%)	9 (2%)
The overutilization of antibiotics can lead to an escalation in bacterial resistance toward these medications	301 (78.4%)	27 (7.1%)	56 (14.5%)
Antibiotics exhibit efficacy in combating bacterial infections	346 (90.1%)	13 (3.4%)	25 (6.5%)
In the event of experiencing a cutaneous response while utilizing an antibiotic, it is advised to refrain from readministering the same antibiotic	276 (71.8%)	104 (27.1%)	4 (1.1%)
Antibiotics expedite the recuperation process for the majority of respiratory infections, like coughs and colds	319 (83.1%)	51 (13.3%)	14 (3.6%)
Antibiotics have been found to exhibit efficacy in combating viral infections	179 (46.6%)	141 (36.7%)	64 (16.7%)
In the event of experiencing adverse effects during a regimen of antibiotic therapy, it is advisable to promptly discontinue their administration	276 (71.8%)	104 (27.1%)	4 (1.1%)

As shown in Table 3, 354 (92.1%) participants agreed that it is important to finish the prescribed antibiotic regimen, and an attitude of refraining from retaining drugs for future utilization was observed in 335 (87.2%). Overall, 346 (90%) participants expressed the belief that getting antibiotics from friends and family without first consulting a physician is not advisable. Additionally, 281 (73.1%) individuals emphasized the importance of obtaining a prescription to purchase antibiotics at a drugstore. However, a significant number of respondents exhibited a negative attitude towards the utilization of antibiotics for the management of sore throat (55.7%) and cough (63.77%). In all, 304 (79.2%) respondents indicated that they had utilized antibiotics within a year before the study period. Among them, 206 (53.5%) participants reported using antibiotics on a single occasion, while 150 (39%) indicated using antibiotics twice, and a very small number of participants (n=29, 7.5%) reported using antibiotics three times. During this time frame, a significant proportion of individuals (n=111, 65.3%) engaged in the practice of self-administering antibiotics without seeking professional medical advice or consultation. Moreover, 350 (91.2%) respondents obtained antibiotics from a pharmacy, while 5.3% and 3.5% of respondents obtained antibiotics from friends and family respectively. When queried about their adherence to the full course of antibiotics, only 202 (66.4%) individuals reported completing the prescribed prescription. The primary rationale cited was a perceived improvement in well-being (n=69, 67.6%), closely followed by instances of forgetfulness (n=20, 19.6%), and the occurrence of adverse effects (n=13, 12.7%).

**Table 3:** Prevailing attitudes toward antibiotic use

	Strongly agree, n (%)	Agree, n (%)	Neutral, n (%)	Strongly disagree, n (%)	Disagree, n (%)
When experiencing symptoms of a sore throat, my personal preference is to utilize an antibiotic treatment	90 (23.4%)	124 (32.3%)	5 (1.3%)	147 (38.3%)	18 (4.6%)
I consistently adhere to the prescribed regimen of antibiotic treatment to its completion, even in instances where symptomatic relief is experienced	310 (80.7%)	44 (11.5%)	0 (0%)	18 (4.7%)	12 (3.1%)
In cases when a cough persists for a duration beyond one week, I am inclined to opt for the utilization of an antibiotic	75 (19.5%)	170 (44.2%)	2 (0.5%)	78 (20.3%)	59 (15.3%)
In certain instances, if I experience an improvement in my condition within a few days, I occasionally discontinue the administration of antibiotics prior to finishing the prescribed treatment regimen	19 (4.9%)	90 (23.5%)	2 (0.5%)	129 (33.6%)	144 (37.5%)
I hold a preference for the accessibility of antibiotics from pharmacies without the requirement of a prescription	18 (4.7%)	83 (21.6%)	2 (0.5%)	143 (37.5%)	138 (35.9%)
The acquisition of antibiotics from acquaintances or family members, bypassing the need for professional medical consultation, is perceived as advantageous	17 (4.4%)	19 (4.9%)	2 (0.5%)	125 (32.6%)	221 (57.6%)
I have a personal inclination towards maintaining a supply of antibiotics within my household, as a precautionary measure for potential future requirements	15 (3.9%)	33 (8.6%)	1 (0.2%)	153 (39.8%)	182 (47.5%)

## Discussion

The objective of this study was to evaluate the level of knowledge, attitudes, and behavior of the general population in Muzaffarpur concerning the utilization of antibiotics. Our findings revealed a widespread prevalence of antibiotics use, and a substantial proportion of individuals acquired them without a valid prescription. The participants demonstrated a general lack of comprehension and a pessimistic attitude toward the proper utilization of antibiotics. Furthermore, occurrences of malpractice were observed, such as instances where people neglected to comply with the recommended dosage or engaged in the unauthorized procurement of antibiotics.

The survey results indicate that a significant proportion of participants (96%) acknowledged the necessity of using distinct antibiotics for treating various diseases. However, a majority of these individuals (83%) held the misconception that antibiotics possess the ability to expedite the recovery process for cough-related ailments. This contrasts with the findings of previous studies from Kuwait and Saudi Arabia, where the reported percentages were 54.4% [18] and 52.2% [19] respectively. The observed disparity may be attributed to variations in sociodemographic characteristics and contextual factors. The prevalence of upper respiratory illnesses may contribute to the misuse of antibiotics, exacerbating the global issue of antimicrobial resistance.

Of note, a significant proportion of participants (78.3%) agreed with the notion that the superfluous utilization of antibiotics has the potential to contribute to the emergence of antimicrobial resistance. Our findings reveal a marginally elevated percentage in this regard compared to the research conducted in Bahir Dar (69.7%) [20], Jordan (50%) [21], and Namibia (72%) [22]. While this observation is very positive, it is worth noting that only approximately one-third (31%) of the participants acknowledged the issue as a global concern. The findings align with the study conducted in Jordan [21], emphasizing the presence of a knowledge gap among users.

Our results indicate a favorable attitude toward the importance of completing the entire course of antibiotics (92.1%) and refraining from utilizing any remaining medication (87.2%). This finding is consistent with a previous study, wherein it was reported that only 17% of respondents stored antibiotics in their households for potential future use [23]. However, the aforementioned study conducted in Namibia reported a far higher percentage of individuals, specifically 28.5%, who stored antibiotics in their households for potential future utilization [24]. Concerning suitable methods of obtaining antibiotics, the participants had a positive disposition. A majority of the respondents agreed with the necessity of consulting a physician before acquiring antibiotics (90%), as well as obtaining a prescription to acquire antibiotics (73.1%). This particular finding exhibits a slightly elevated percentage in comparison to a study conducted in Saudi Arabia, in which reported figures were 76.6% and 66.6%, respectively [18].

In the present investigation, almost 79% of the participants indicated that they had utilized antibiotics at least once over the 12-month period before the study. This report exhibits a similarity to a study conducted in Namibia, with a correlation of 80% [22]. However, the proportion of research participants in a study conducted in Lithuania was found to be 24.9% [24], whereas it was 35.9% in Bahir Dar [25]. Several factors may contribute to the observed disparity, such as the distribution of disease prevalence in the specific region and the duration of data collection. For instance, upper respiratory illnesses, for which patients frequently resort to antibiotic usage, exhibit seasonal fluctuations. Conversely, it might be contended that the impact of antibiotic accessibility lies in the potential for increased usage among individuals, as convenient availability may incentivize patients to use antibiotics for small ailments.

Our findings suggest that around 65% of antibiotic usage occurs without a valid prescription. This is intriguing, considering a majority of the participants expressed a belief in the necessity of obtaining a prescription to acquire antibiotics. This discrepancy may suggest that factors other than the knowledge and beliefs of users play a role in their actual practices. Furthermore, a notable degree of variance was observed across the literature about the practice of antibiotic use without a prescription. For example, a study conducted in India indicated that almost 76% of antibiotic usage occurred without a prescription [26]. Similarly, in Italy, the percentage was found to be 32.7% [27], while it was 28.8% in Saudi Arabia [19]. In Hong Kong, the rate of antibiotic usage without prescription was estimated to be 9% [28]. The observed discrepancy may be attributed to variations in regulatory frameworks and their enforcement across different regions.

In terms of where antibiotics came from, drug stores accounted for 91.2% of all sources. The majority of participants in this study admitted to using antibiotics without a prescription. This study suggests the widespread distribution of antibiotics without a prescription. As a result, it is essential to consider this problem from the perspective of pharmacy specialists and take the right steps to curb it, such as enacting stronger rules on the distribution of pharmaceuticals without a prescription. Another area where the participants in our study showed deficiency was in completing the duration of the regimen. In this study, 34% or so of the participants did not finish the antibiotic treatment. Fortunately, this figure is lower than that in research conducted in China (49.8%) and Malaysia (55.9%) [29,30]. However, It is significantly larger compared to the figures from Namibia (20%) [22]. Several studies have reported this improper practice and linked it to a rise in antibiotic resistance [4,5].

This study has several limitations. Firstly, it relied on self-reported data, which may introduce recall and social desirability biases, potentially affecting the accuracy of responses related to antibiotic usage. Additionally, the study's focus on a specific geographic area, a city in Bihar and its surrounding regions, limits the generalizability of its findings to broader populations. Furthermore, the cross-sectional design limited our ability to establish causal relationships between variables, and the data represents a snapshot in time, potentially missing long-term trends or changes in behavior. Despite these limitations, the study provides valuable insights into attitudes about antibiotic use and highlights the need for educational and regulatory interventions among the general public.

## Conclusions

This study revealed a prevalent utilization of antibiotics among the general public in Muzaffarpur, with a significant proportion of these medications being obtained without a prescription. The respondents exhibited a general lack of awareness, particularly regarding the significance of antibiotics in the treatment of mild viral infections They had a negative outlook towards antibiotic use for the treatment of cold and sore throat symptoms. Additionally, notable instances of improper practices were observed, including individuals failing to adhere to the prescribed dosage and engaging in the acquisition of antibiotics without a valid prescription. Therefore, it is crucial to implement educational interventions focusing on the utilization of antibiotics and its correlation with the emergence of drug resistance to promote the prudent and responsible use of these medications. Implementing nationwide antibiotic laws is a crucial strategy for mitigating the prevalence of over-the-counter sales, thereby curbing the practice of self-prescription of antibiotics.
